# Photon-Counting Detector Computed Tomography (PCD-CT): A New Era for Cardiovascular Imaging? Current Status and Future Outlooks

**DOI:** 10.3390/jcdd11040127

**Published:** 2024-04-21

**Authors:** Pietro G. Lacaita, Anna Luger, Felix Troger, Gerlig Widmann, Gudrun M. Feuchtner

**Affiliations:** Department Radiology, Innsbruck Medical University, Anichstrasse 35, 6020 Innsbruck, Austria; placaita@gmail.com (P.G.L.); anna.luger@i-med.ac.at (A.L.); felix.troger@i-med.ac.at (F.T.); gerlig.widmann@tirol-kliniken.at (G.W.)

**Keywords:** cardiovascular diseases, imaging, computed tomography (CT), coronary computed tomography angiography (CTA), photon-counting detector CT (PCD-CT)

## Abstract

Photon-counting detector computed tomography (PCD-CT) represents a revolutionary new generation of computed tomography (CT) for the imaging of patients with cardiovascular diseases. Since its commercial market introduction in 2021, numerous studies have identified advantages of this new technology in the field of cardiovascular imaging, including improved image quality due to an enhanced contrast-to-noise ratio, superior spatial resolution, reduced artifacts, and a reduced radiation dose. The aim of this narrative review was to discuss the current scientific literature, and to find answers to the question of whether PCD-CT has yet led to a true step-change and significant progress in cardiovascular imaging.

## 1. Introduction

Coronary computed tomography (CT) angiography (CTA) is one of the central imaging modalities for the evaluation of patients with cardiovascular diseases, in particular, coronary artery disease, but also congenital heart disease.

Coronary CTA is rated as a Class I b indication for the assessment of patients with chronic coronary syndromes (CCS) and low-to-intermediate pre-test probability according to the European Society of Cardiology (ESC) guidelines 2019 [1]. The American Heart Association (AHA) 2021 Chest Pain Guideline also classifies coronary CTA as a Class I indication for patients with an intermediate (15–75%) pre-test probability of coronary artery disease (CAD) [2]. Coronary CTA allows for the quantification of coronary stenosis and has shown equivalent outcomes to invasive coronary angiography (ICA) in a large international prospective multicentric pragmatic randomized trial (DISCHARGE) [3]. Beyond this, CTA enables the characterization of plaque morphology. Lipid-rich necrotic-core low-attenuation plaque (LAP) can be detected based on the presence of a hypoattenuating plaque component with less than 30 Hounsfield Units (HUs) [4]. Fibro-fatty plaques are defined as hypoattenuating lesions with less than 130 HUs, and calcified plaques as hyperattenuating lesions with densities higher than 130 HUs. Lipid-rich necrotic (LN)-LAP is a prognosticator for adverse cardiovascular outcomes, as shown by the prospective randomized SCOT-Heart trial. Beyond LN-LAP, three other “high-risk-plaque” features have been identified in comparative studies with histopathology: spotty calcification (less than 3 mm calcified nodules), positive plaque remodeling (outside of the vessel lumen), and the Napkin-Ring sign [5,6]. These imaging characteristics point to “vulnerable” plaque, more prone to rupture and causing acute coronary syndromes (ACS). Importantly, higher plaque density has been linked with more “stable plaque”, with a linear relationship. A very low risk of ACS in highly dense plaque higher than 1000 HU (“1k plaque”) has been demonstrated by the ICONIC investigators [7].

However, highly dense calcified plaques cause varying degrees of “blooming” artifacts, overshadowing the coronary lumen. This is one of the major limitations of coronary CTA to date, resulting in an overestimation of coronary stenosis compared to invasive angiography. Both specificity and positive predictive value decline linearly with higher coronary calcium load, quantified by the Agatston score (AU). In particular, at an AU of more than 400 HU, the accuracy of CTA for stenosis grading is significantly lower [8].

With the introduction of a new generation of computed tomography (CT) technology, photon-counting computed tomography (PCD-CT), an array of new possible applications has emerged, and this technology may also have the potential to overcome the current shortcomings of conventional CT.

Photon-counting detector CT (PCD-CT) is based on novel advancements and has become widely commercially available since its market introduction in November 2021.

### 1.1. Technical Principles of Photon-Counting Computed Tomography (PCD-CT)

PCD-CT utilizes an innovative detector technology distinct from conventional CT [9,10,11,12,13,14]. Nowadays, most commercially available CT detectors employ standard energy-integrating detectors (EIDs), which utilize an indirect dual-step conversion process. In EIDs, incidental X-ray photons are converted into visible light by a scintillator, and then, a photodiode array converts this light into electrical signals. Consequently, the detector integrates the energy from all X-ray photons within a set duration, losing the ability to distinguish individual photon energies. Beyond this, EID applies a smaller weight to lower-energy photons, which contain a larger amount of soft tissue contrast information than higher-energy photons, resulting in lower and often suboptimal soft tissue contrast [12].

Additionally, conventional EIDs utilize septa layers to separate each detector cell, causing significant disadvantages such as limitations in spatial resolution and increased scattering; thus, EIDs add electronic noise and Swank noise. All of these factors result in increased image noise [12]. Hence, the current limitations of EID-CT include (1) low and often insufficient soft tissue contrast; (2) a lack of tissue-specific information in the images; and (3) a relatively high radiation dose [12].

**Figure 1:**
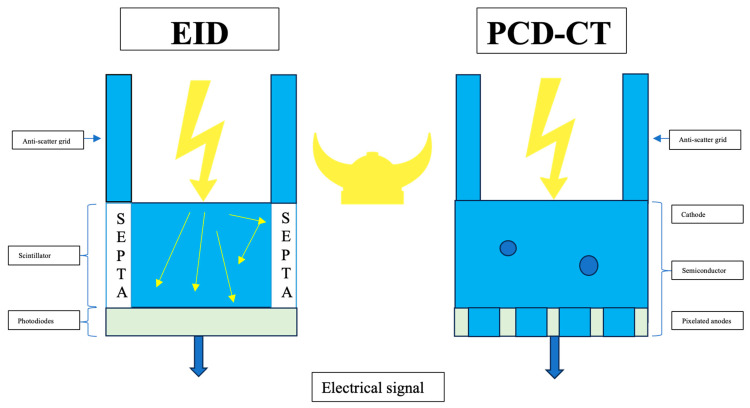
Technical principles of photon-counting detector CT (PCD-CT) compared to those of energy-integrated detector (EID)-CT. On the left side, a standard EID-CT is illustrated. EID-CT is characterized by the use of a scintillator and septa. On the right side, a PCD-CT that directly converts and counts X-ray photons into electrical signals without the need for scintillator layers is shown.

In contrast, PCD-CT uses semiconductor detectors made of materials such as cadmium telluride, cadmium zinc telluride, or silicon. These detectors directly convert X-ray photons into electrical signals without the need for scintillator layers, resulting in a significant reduction in electronic noise (Figure 1); thus, electronic and Swank noise do not change the output signal intensity (i.e., the counts) [12].

PCD-CT detectors consist of individual small detector elements that are not separated by septa, allowing them to measure single photons. These new detectors pair a semiconductor sensor with a readout circuit. When a photon interacts with the sensor, it generates electron–hole pairs, which move towards the electrodes due to a bias voltage applied to the sensor. The pixels collect the charge carriers, producing an electrical signal proportional to the photon’s energy. Furthermore, PCD-CT allows for categorizing incoming photons based on their energy, enabling the filtering out of electronic noise. PCD-CT offers the advantage of operating in two distinct modalities: conventional and spectral imaging.

Compared to conventional multidetector CT, PCD-CT offers a higher spatial resolution (0.16–0.2 mm^2^) and up to 40 lp/cm [12], which is close to the resolution obtained by flat-panel invasive coronary angiography. Furthermore, a higher temporal resolution (up to 66 ms) is maintained compared to dual-source CT (DSCT), due to a similar gantry rotation speed of ≥0.25 s.

Second, PCD-CT provides multienergetic image reconstruction (“spectral imaging”) from one detector, in contrast to conventional dual energy CT (DECT) using two X-ray sources (“dual source” CT). DECT utilizes two distinct X-ray tubes and detectors positioned at a 90-degree angle, allowing for simultaneous image acquisition at multiple energy levels.

Spectral imaging, defined as multienergetic imaging, has become possible due to the dual-source geometry of the scanner and offers advantages such as improved image quality and the possibility of multienergetic subtraction, allowing for the decomposition of different materials based on their individual X-ray absorption. For example, high-quality iodine maps can be generated, or other materials (such as calcium or monosodium urate crystals) can be extrapolated.

In contrast to DECT, PCD-CT permits “k-edge” imaging by separating specific materials based on their atomic energy thresholds (lower and higher energy of the k-edge). Subsequently, various distinct types of contrast agents (such as bismuth, gold, platinum, silver, and ytterbium) can be differentiated according to their unique K-edges. Even new types of contrast agents, including nanoparticles tailored to specific cells or enzymes, might be used, which would broaden the spectrum of imaging from pure anatomical to functional and molecular [12]. However, to date, these agents have only been tested in animal studies and not in humans [14].

Furthermore, PCD-CT scanners offer another technical advantage, the ultra-high-resolution (UHR) mode.

In summary, the technical advantages of PCD-CT include a reduction in electronic noise at the same radiation dose compared to EID, and a soft tissue contrast improvement. PCD-CT weighs all photons equally; hence, it gives relatively more weight to low-energy photons compared to EID-CT, which provides more contrast information [14].

### 1.2. Rationale and Knowledge Gap

PCD-CT is a novel CT technology which has only been commercially available for 2 years. To date, over 300 publications using PCD-CT have been published, including several reviews [9,10,11,12,13,14], with an ongoing increase in the monthly publication rate. Therefore, a contemporary narrative review discussing the latest publications on the clinical potential of PCD-CT is warranted.

### 1.3. Objectives

The objectives of this study are to review the current scientific literature and discuss the potential of the new commercially available PCD-CT scanner types for imaging of patients with cardiovascular diseases, such as coronary heart disease, or structural heart disease, and to discuss whether PCD-CT may lead to advances in meeting the clinically relevant requirements of cardiovascular imaging.

## 2. Materials and Methods

A literature review of scientific papers investigating PCD-CT for cardiovascular applications was performed by using Pubmed.central and google.com searches. The following key words were searched: photon-counting computed tomography (PCCT), photon-counting detector computed tomography (PCD-CT) and/or coronary arteries, coronary computed tomography angiography (CTA), coronary stents, coronary stenosis. The current scientific evidence is summarized and discussed critically, as well as future potential clinical applications. The advantages and capabilities of PCD-CT technology are described and illustrated.

## 3. Results/Discussion

### 3.1. Does PCD-CT Have Potential to Reduce “Calcium Blooming” and Improve the Accuracy of Coronary Stenosis Grading?

Artificial calcium blooming in coronary CT-angiography (CTA) is a significant challenge in accurately assessing stenosis severity. It often leads to the overestimation of stenosis [8], followed by the increased use of invasive imaging procedures such as catheter angiography. Based on the two major technical advantages of PCD-CT (higher spatial resolution and spectral imaging) [9,10,11,12,13,14], calcium blooming should be reduced, and as a result, the overestimation of coronary stenosis might be diminished.

In a recent study, Wolf et al. [15] investigated the quantification of coronary artery stenosis in a phantom as well as in an in vivo series of 33 patients. Their findings revealed that blooming artifacts and stenosis severity decreased for calcified and mixed plaques with increasing virtual monoenergetic imaging (VMI) levels. Furthermore, PCD-CT allows for the separation of imaging for iodine and calcium, enabling virtual non-calcium reconstruction.

PCD-CT offers a specific spectral imaging option that utilizes an automated reconstruction algorithm for the visual subtraction of calcium from the coronary vessel wall (PureLumen^TM^), which represents a promising advancement for imaging the coronary arteries.

Only one phantom study, conducted by Allmendinger et al. [16] in 2022 using a moving device, has validated this algorithm, showing encouraging results. In this study, two different iodine-contrast vessel phantoms with variable degrees of diameter stenosis (25% and 50%) but consistent keV values were employed. The authors demonstrated that a closer approximation of genuine stenosis grade compared to virtual monoenergetic images was attained, and that blooming artifacts were greatly minimized. Remarkably, there as a substantial difference between monoenergetic image reconstructions and PureLumen^TM^ for both lesion sizes (25% and 50%) (*p* < 0.05).

Additionally, the automated calcium-removal algorithm maintained the accuracy of stenosis grading even in the presence of motion, with heart rates of up to 80 beats per minute.

Overall, the innovative calcium-removal image reconstruction algorithm shows promise for improving coronary vessel imaging in clinical practice. Nishihara et al. [17] recently published a retrospective study involving 17 patients, each presenting with at least one significant coronary stenosis (≥50%) with identified calcified plaque. These patients underwent photon-counting CT followed by invasive coronary angiography (ICA) within 60 days of coronary CTA. The reconstructed images with calcium-removal and the conventional images were compared with the reference ICA images. Calcium-removal images were generated from conventional images, and both sets of images were assessed against the ICA images, serving as the reference standard. The results showed that the calcium-removal images significantly outperformed the conventional images in terms of accuracy, specificity, and positive predictive value, (80.5% vs. 69.5%, *p* = 0.002, and 64.1% vs. 52.0%, *p* < 0.001, respectively).

However, additional in vivo research in larger cohorts is required to validate its performance and clinical benefits.

The first in vivo investigation, involving 30 patients, was published recently by Mergen et al. [18], who showed that the virtual non-contrast calcium (VNCa)-removal algorithm improved the image quality and the quantification of calcified, mainly minimal or moderate stenosis. Of note, only 12% of calcified lesions could not be analyzed and were excluded. The concordance of n = 71 stenosis categories between quantitative coronary angiography (QCA) and virtual monoenergetic imaging (VMI) was good, with values of 69% (kappa, k = 0.70) and 62% (k = 0.70); however, these values improved to 79% (k = 0.82) and 87% (k = 0.89) when using VNCa images for readers 1 and 2, respectively. Notably, 27% and 37% of stenoses were overestimated utilizing VMI, compared to only 7% and 8% of stenoses when assessing stenosis on VNCa images for readers 1 and 2, respectively.

In a recent study by Haag et al. [19], virtual non-contrast (PureCalcium^TM^) image reconstructions were compared in 170 patients for measuring coronary artery calcium (CAC) Agatston scores with conventional unenhanced CAC scores. There was a good correlation in calcium scores between TNC and PureCalcium (kappa, k = 0.88); however, limited agreement was found in Bland Altman Plots, especially for patients without plaque burden who were misclassified (*p* < 0.001), with an overestimation of CAC scores known from prior studies, which should be taken into consideration in clinical practice. More investigations in larger cohorts are required to evaluate the performance of VNCa in clinical practice.

Another study by Hagar et al. [20] evaluated the accuracy of UHR coronary CTA in 68 patients with severe aortic stenosis and a high-risk coronary disease profile compared to ICA. The image quality and the accuracy of UHR CTA in detecting CAD were excellent, with AUCs of 0.93 per patient, 0.94 per vessel, and 0.92 per segment. The sensitivity, specificity, and accuracy, respectively, were 96%, 84%, and 88% per patient (n = 68); 89%, 91%, and 91% per vessel (n = 204); and 77%, 95%, and 95% per segment (n = 965). In particular, the UHR mode has the potential to improve accuracy for stenosis grading, which was recently investigated by Koos et al. (refer to Section 3.5 below) [21].

Halfmann et al. [22] performed an in vitro investigation and used an in vivo sample including 114 patients. The in vitro DS percentage measurements were more accurate with increasing spatial resolutions for both 25% and 50% stenosis. The in vivo results confirmed a decreasing median DS percentage with increasing spatial resolution for calcified stenoses (n = 161) (standard resolution, high spatial resolution, and ultra-high spatial resolution, respectively: 41.5%, 34.8%, and 26.7%, *p* < 0.001), whereas non-calcified and mixed plaques did not show a difference. Ultra-high-spatial-resolution reconstructions led to the reclassification of 54.4% of patients into the lower stenosis severity (CAD-RADS) category, which was more than the percentage assigned using standard resolution.

In conclusion, both in vivo and in vitro coronary stenosis assessments are improved for calcified stenoses by using ultra-high-spatial-resolution PCD-CT reconstructions and clinically relevant rates of reclassification.

### 3.2. Is the Evaluation of Coronary Stents More Accurate with PCD-CT?

**Figure 2:**
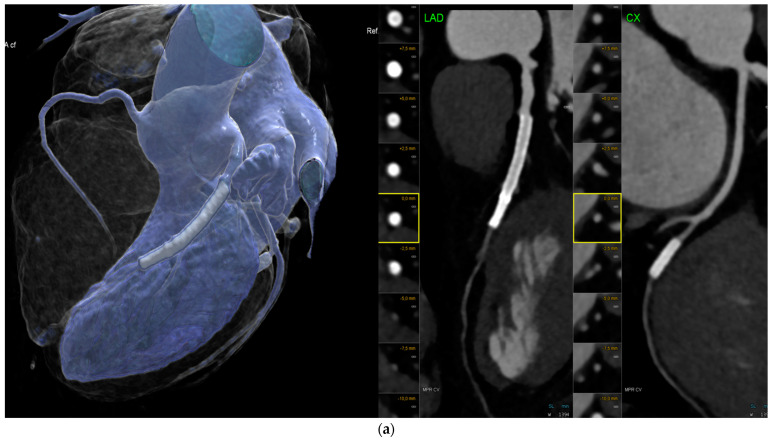

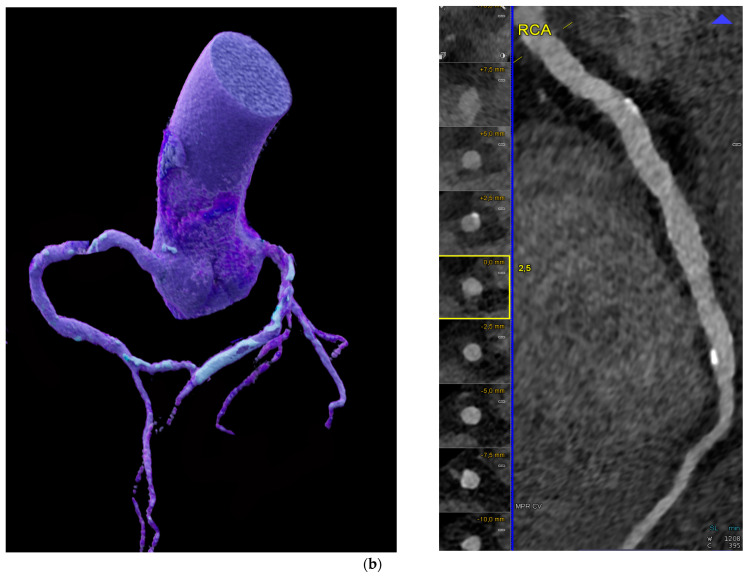
(**a**). Imaging of coronary stents by conventional energy-integrating detector (EID)-CT: Multiple left anterior descending (LAD) coronary stents with inadequate in-stent lumen visualization of the distal LAD and circumflex artery (CX) stent due to artifacts. Left: volume-rendering technique (VRT) and right: curved multiplanar reformations (cMRP) of the LAD and CX. (**b**). Photon-counting detector CT (PCD-CT): Curved multiplanar reformation (cMRP) (left) shows calcified right coronary artery (RCA) in a 76-year-old male patient with a high calcium load, but reduced blooming artefacts with less than 50% stenosis. Stent in the mid-left anterior descending (LAD) proximal coronary artery (VRT, right panel) and heavy vessel wall calcification. VRT = volume-rendering technique.

For the assessment of stent patency, the accuracy of conventional EID-CT is constrained. Coronary CTA allows for the evaluation of proximal and larger stents with a diameter of ≥3 mm; however, smaller stents (Figure 2a,b) and special stent types, depending on the stent strut thickness and material composition, are typically not able to be evaluated reliably, due to artifacts from stent struts limiting the visualization of the in-stent lumen (ISL). In-stent restenosis (ISR) of greater than 50% was detected with a sensitivity of 90% and a specificity of 94%, according to a meta-analysis by Dai et al. (2018) including 35 studies and 4131 stents.

The pooled positive LR and negative LR were 14.0 and 0.1, which are at the lower end of being diagnostically acceptable. Stents with thick struts (≥100 μm) and bifurcation stents were frequently non-assessable [23]. However, Wan et al. [24] found that in a comparative study between CTA and invasive coronary angiography (ICA) in 318 stents, the positive predictive value (PPV) of CTA for the detection of ISR was only moderate, at 67% per stent.

Mannil et al. [25] investigated 18 coronary stents in vitro using PCD-CT compared to conventional EID. The purpose of this series was to investigate the potential benefits of PCD-CT. Consequently, for both 0-degree and 90-degree phantom positions, the in-stent diameter was 28.8% and 8.4% larger in PCD-CT (0.85 vs. 0.83 mm) vs. EID (0.66 mm vs. 0.76 mm), respectively. With PCD-CT, there was less image noise and a smaller artificial increase in in-stent attenuation (*p* < 0.001), indicating improved quantitative image quality. Additionally, the subjective in-stent visibility was superior for PCD-CT (*p* < 0.001) [25]. PCD-CT provided the highest in-plane resolution of 0.11 mm and a through-plane resolution of 0.16 mm when the ultrahigh-resolution modus (UHR) was applied. These studies were the first to show promising results for the improved in vivo visualization of coronary stents.

Boccalini et al. [26] demonstrated, for the first time, in a preliminary in vivo study of 8 patients, the improved visualization of stents. Both the objective and subjective image quality parameters were improved, and the radiation dose was reduced.

In another in vivo study [27], Geering et al. compared different image reconstruction algorithms regarding their performance for ISL visualization. The ISL diameters were largest for a 0.2 mm slice thickness and the Bv72, Bv80, and Bv89 kernels. Individual adaptations to patients’ characteristics (such as body mass index) must be taken into consideration.

Further, a higher temporal resolution of ≥66 ms was found to improve the visualization of coronary stents in vivo in a series of 30 patients [28] due to fewer motion artifacts, superior vessel delineation and in-stent lumen visualization, fewer stent blooming artifacts, and superior vessel and stent sharpness.

Most recently, Hagar et al. [29] conducted a comparison of the accuracy of PCD-CT and ICA for the detection of ISR > 50% in 44 stents. Even though there were only five cases with ISR, they all could be detected by PCD-CT (100% accuracy).

In summary, PCD-CT offers promising potential for the visualization of coronary stents and the detection of ISR. However, more in vivo studies in larger cohorts, validating the accuracy of PCD-CT compared to ICA, are required.

### 3.3. Clinical Efficacy Studies

#### 3.3.1. Can PCD-CT Reduce Referrals for Invasive Coronary Angiography (ICA) ?

There is currently only one published clinical efficacy study: Simon et al. [30] recently compared the severity of coronary stenosis stratified by CADRADS in two groups of 401 patients, totaling 802 individuals. One group was scanned with EID-CT, while the other underwent PCD-CT. According to the study, there was a 50% decrease in the number of patients referred for invasive catheter angiography (ICA), while more cases of obstructive CAD were reported in the EID-CT group.

The ultra-high-resolution mode, as demonstrated by recent studies, improves the grading of coronary stenosis and the visualization of coronary plaques [21,22].

#### 3.3.2. Is PCD-CT Capable of Decreasing the Amount of Contrast Agent Applied?

Other benefits of adopting PCD-CT include reduced contrast agent volume due to shorter scan times and spectral image reconstruction (Table 1).

Emrich et al. [31] demonstrated, in a phantom investigation using a 3D-printed model, that the contrast medium volume could be lowered while maintaining good image quality. Their findings indicate that virtual monoenergetic reconstructions at 40 keV could reduce contrast medium concentrations by 50% while maintaining diagnostic quality in coronary CT angiography. Contrast medium volume savings in PCD-CT were analyzed in a recent in vivo coronary CT angiography study by Cundari et al. [32] involving a total of 100 patients. The results showed that PCD-CT allows for a significant reduction in contrast volume of 20% without compromising image quality. Higashigaito et al. [33] conducted a prospective investigation on a low-volume contrast protocol for thoracoabdominal CTA in 100 patients who underwent both PCD-CT and EID-CT at equal radiation doses. The final results demonstrated that adopting a low-volume contrast agent protocol with PCD-CT reduced the contrast volume by 25% (52.5 mL), while the image quality remained comparable to EID-CT at the same radiation dose. VMI at 50 keV provided the best image quality trade-off, with a 25% higher contrast-to-noise ratio (CNR) compared to EID-CT.

The reduction in contrast volume is a significant advantage in clinical practice, particularly for patients with renal dysfunction and other comorbidities. Langenbach et al. [34] conducted a study utilizing a dual-energy spectral detector and virtual monoenergetic imaging reconstruction in 60 patients undergoing pre-interventional transcatheter aortic valve planning. The results revealed that reducing the contrast volume to 40 mL did not degrade image quality. Both the signal-to-noise ratio (SNR; −1.1, *p* = 0.726) and the contrast-to-noise ratio (CNR; 0.0, *p* = 0.999) were comparable. Furthermore, 40-keV virtual monoenergetic image (VMI) reconstructions outperformed standard reconstructions, with a significantly greater SNR (+6.04, *p* < 0.001).

#### 3.3.3. Does PCD-CT Permit Lowering Radiation Exposure?

Pinos et al. [35] compared the image quality of coronary CTA in 20 obese patients (mean BMI, 28.5 kg/m^2^) using PCD-CT and EID-CT. The authors found that PCD-CT provided superior image quality, with higher contrast and less noise, particularly in obese patients (all *p* < 0.008) with a BMI over 30 kg/m^2^. A lower computed tomography dose index (CTDI) volume was reported for PCD-CT (33.1 vs. 25.2, *p* = 0.38), although this difference was not significant. By using only a contrast volume of 40–60 mL, adequate intraluminal vessel enhancement was obtained (T3D: 363 HU). The mean CT attenuation of the coronary arteries decreased markedly with increasing keV levels, from 994 HU at 40 keV to 408 keV at 70 keV, which must be taken into consideration for the selection of keV levels for coronary CTA image analysis.

Another study regarding image quality and contrast medium reduction in obese patients was conducted in 2023 by Hagen et al. [36]. In this study, the authors compared 68 overweight patients who underwent a portal venous contrast-enhanced abdomen CT with a commercial first-generation photon-counting CT and had undergone a previous examination on a second-generation dual-source energy-integrating detector CT. The readers analyzed the image noise, contrast-to-noise ratio (CNR), and signal-to-noise ratio (SNR). The results demonstrated that the image quality remained equivalent despite a significant reduction in contrast volume. Additionally, the radiation dose was decreased by 27% and 34% for DLP, and by 31% for CTDI volume for abdominal contrast-enhanced CT, respectively.

Graafen et al. [37] published an article comparing the radiation dose and image quality of chest CT scans conducted with PCD-CT and EID-CT in a group of 32 patients. The results were significant: the mean CTDI vol was 2.0 times higher in the conventional EID-CT scans (1.8 ± 0.5 mGy) compared to PCD-CT (0.9 ± 0.5 mGy, *p* < 0.001). Despite the reduction in CTDI, there were no noticeable differences in noise and SNR.

Finally, Euler et al. [38] compared the image quality of high-pitch CTA between PCD-CT and EID-CT in 40 patients undergoing thoracoabdominal aorta angiography. The contrast-to-noise ratio (CNR) was significantly higher for 40 and 45 keV of PCD-CT compared to EID-CT. The CNR gain of PCD-CT rose by 34% in overweight patients compared to normal-weight patients.

**Table 1:** The clinical efficacy of photon-counting detector computed tomography (PCD-CT)—In vivo studies.

	N (Patients)	Specification
VNC calcium removal (Purelumen^TM^)		
Mergen et al. [18]	30	* Accuracy in coronary stenosis grading improved * Image quality improved
Wolf et al. [15]	33 (+in vitro phantom)	Stenosis severity and blooming artifacts decreased
STENTS—in vivo studies		
Hagar et al. [20]	44 (n = 5 ISRs)	5 (100%) ISRs detected vs. ICA
Contrast agent reduction		
Cundari et al. [32]	100	20%
Higashigaito et al. [33]	100	25% (52.5 mL)
Radiation dose reduction		
Hagen et al. [36]	40 (obese)	31% CTDI vol
Graafen et al. [37]	32	2 times less CTDI vol (*p* < 0.001)
Accuracy of stenosis grading (UHR mode)		
Koons et al. [21]	23 patients 34 lesions	* Stenosis severity reduced in PCD-CT (*p* < 0.001): 11% reduction in percent diameter stenosis (%DS) * 15/34 underwent changes in stenosis severity
Halfmann et al. [22]	114 patients (+in vitro phantom)	* Calcified lesions led to more accurate stenosis % * NCP and mixed equal

Abbreviations: ISR = in-stent restenosis. ICA = invasive coronary angiography. mL = milliliter. CTDI = computed tomography dose index. Vol = volume. PCD-CT = photon-counting detector computed tomography. NCP = non-calcified plaque.

### 3.4. Are There Further Clinical Applications of Spectral Imaging?

**Figure 3:**
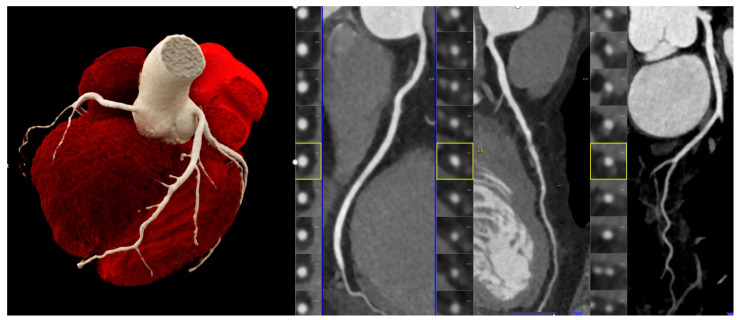
Coronary CTA: Optimal image quality with low image noise and high contrast resolution allows for the reliable exclusion of coronary artery disease. Left: 3D-VRT and right: curved multiplanar reformation (cMPR) of RCA, LAD, and CX arteries show absence of coronary plaque and stenosis.

PCD-CDT not only permits the reliable exclusion of coronary artery disease (Figure 3) and the grading of coronary stenosis and plaque imaging, but also provides a comprehensive evaluation of the myocardium, cardiac chambers, and cardiac function. Myocardial scar tissue and fibrosis, as well as myocardial extracellular volume (ECV), can be quantified. Aquino et al. [39] published a study last year involving 29 patients who underwent both cardiac CT and magnetic resonance imaging (MRI), using three different techniques: single-energy PCD-CT, dual-energy PCD-CT, and MRI T1 mapping. The results were excellent, demonstrating that both single- and dual-energy PCD-CT exhibited an excellent correlation of the ECV with MRI (r = 0.82 and 0.91, both *p* < 0.001) and showed high reliability with only very minor overestimation and underestimation compared to MRI.

**Figure 4:**
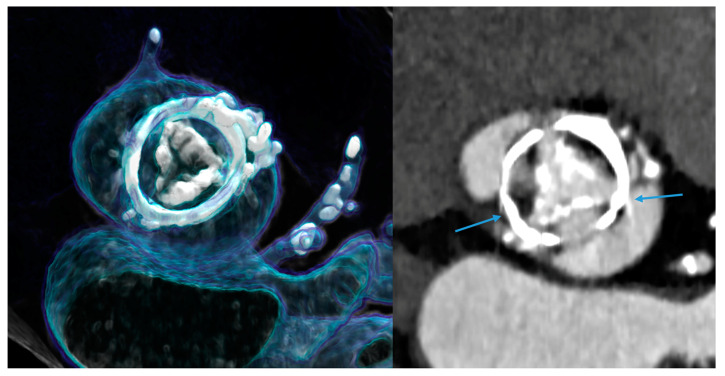
Prosthetic heart valves (PHV) causing metal artifacts. An example of a bioprosthetic valve with an outer stent is shown, causing the blooming of metal and streak artifacts (right, blue arrows). This bioprosthetic valve is also showing signs of degeneration (severely calcified leaflets, inside the stent) 10 years after implantation. Left: 3D-VRT and right: axial MPR.

In addition to this, spectral imaging also permits a reduction in metal artifacts, depending on the material. Artifacts from both stents and other cardiac devices, such as prosthetic heart valves (Figure 4), pacemakers, or other surgical material, can be reduced [40].

In an in vitro study using a multienergy phantom [40], metal artifacts were most prevalent for the steel insert (12.6% average artifacts), followed by titanium (4.2%) and aluminum (1.0%). The strongest metal artifact reduction was noted for the iterative metal artifact reduction algorithm (iMAR) with iMAR and without iMAR (1.4% and 10.5%, respectively; *p* < 0.001), or with virtual monoenergetic images (VMI: 110 keV 2.6% to 150 keV 3.3%, T3D: 11.0%, and none: 16.0%; *p* < 0.001). The best results were achieved when combining iMAR and VMI at 110 keV, while alternating tube potentials (120 kV: 6.6%, 140 kV: 5.2%; *p* = 0.33) and reconstruction kernels (Br36: 5.5%, Br56: 6.4%; *p* = 0.17) was less sufficient. These data indicate a potential significant reduction in artifacts from prosthetic heart valves (Figure 4).

### 3.5. Can the Ultra-High Resolution (UHR) Mode Be Implemented in Clinical Practice?

The UHR mode provides the highest spatial resolution (0.2 mm^2^). As a result, it is expected to reduce the blooming of calcified plaques. Also, the visualization of small-sized vessels could be improved. The superior spatial resolution, however, increases image noise, adversely impacts SNR, and may worsen the visualization of non-calcified plaques. A few studies so far have investigated the UHR mode using a PCD-CT scanner.

The first in vivo quantitative plaque characterization with ultra-high-resolution coronary PCD-CT was published by Mergen et al. [41], who analyzed 22 plaques from 20 patients. The study demonstrated that using different slice thicknesses influenced UHR scanning with regard to quantitative plaque characterization. Furthermore, the reduction in blooming artifacts helped to distinguish fibrotic and lipid-rich plaque components.

Koons et al. [21] examined the severity of coronary stenosis in 23 patients with 34 calcified lesions with PCD-CT and EID-CT. For the UHR Mode, images with a 120 × 0.2 mm detector collimation were acquired. Stenosis was significantly reduced in PCD-CT compared to EID-CT (*p* < 0.001), yielding in an average 11% reduction in percent diameter stenosis. Among the 34 lesions, 15 were reclassified for stenosis severity, with 13 of 34 stenoses being downgraded, potentially sparing patients from unnecessary intervention.

Beyond this, the UHR mode using PCD-CT was also compared to conventional dual-source CT high-pitch scanning (HPS) in 64 patients prior to transcatheter aortic valve implantation (TAVI) for annulus sizing [42]. UHR imaging was found to provide superior image quality in patients with poor image quality using HPS; however, the radiation dose was higher. Both the UHR and HPS techniques produced strongly correlative and accurate values for annulus sizing in the majority of patients (91%).

In summary, the UHR mode requires further prospective studies with larger cohorts to validate its superiority and clinical advantages compared to conventional CT scanning.

Limitations of photon–counting CT: First, the image quality in PCD-CT is restricted by the X-ray tube, which has an insufficient focal spot size. Second, the spectral properties of PCD-CT require a broader energy spectrum in order to fully benefit from all the advantages, such as dose reduction and improved image quality.

Further, there are two contradicting factors posing a technical challenge: pulse pileup and spectral response (due to, e.g., charge sharing). Thus, building a PCD-CT system involves more than swapping the detectors and requires significant effort in other fields as well [12].

Additionally, increased occurrence of step-artifacts has been observed. Therefore, a specific algorithm for automated artificial intelligence (AI)-enabled compensation has been developed and introduced recently. Beyond this, PCD-CT technology is associated with higher costs than conventional CT scanners, which results in financial challenges for the public healthcare sector.

## 4. Conclusions

In summary, PCD-CT has led to a true step-change and significant progress in cardiovascular imaging over EID-CT. The following clinically relevant improvements were noted: higher spatial resolution; reduced calcium blooming; higher accuracy of stenosis grading, especially for severely calcified lesions; improved visualization of the coronary in-stent lumen; a reduction in contrast volume ranging from 25 to 50%; a reduction in radiation exposure of about 30% and up to two-fold; and a reduction in referrals to ICA in one larger cohort.

### Future Outlooks

Prospective studies, preferably multicentric studies enrolling larger cohorts, are required to investigate the full range of clinical benefits and the medical impact of PCD-CT, as well as the accuracy of novel tools such as automated calcium removal. Accordingly, PCD-CT has the potential to overcome the current limitations and shortcomings of EID-CT. Because of its technical superiority, the number of invasive procedures is expected to be reduced, which would not only lead to benefits for individual patients by exposing them to lower procedural complication risk, but would also be cost-effective and place a lower economic burden on currently stretched healthcare systems. Minimizing radiation exposure and contrast medium volume are significant advantages for patient-centered care.
